# Supplementary material for: Multiplexed characterization of rationally designed promoter architectures deconstructs combinatorial logic for IPTG-inducible systems
Source: Nat Commun. 2021 Jan 12;12:325. doi: 10.1038/s41467-020-20094-3 (PMC7804116; doi:10.1038/s41467-020-20094-3)
Supplement: Supplementary file 1 — Supplementary Information [file 41467_2020_20094_MOESM1_ESM.pdf]

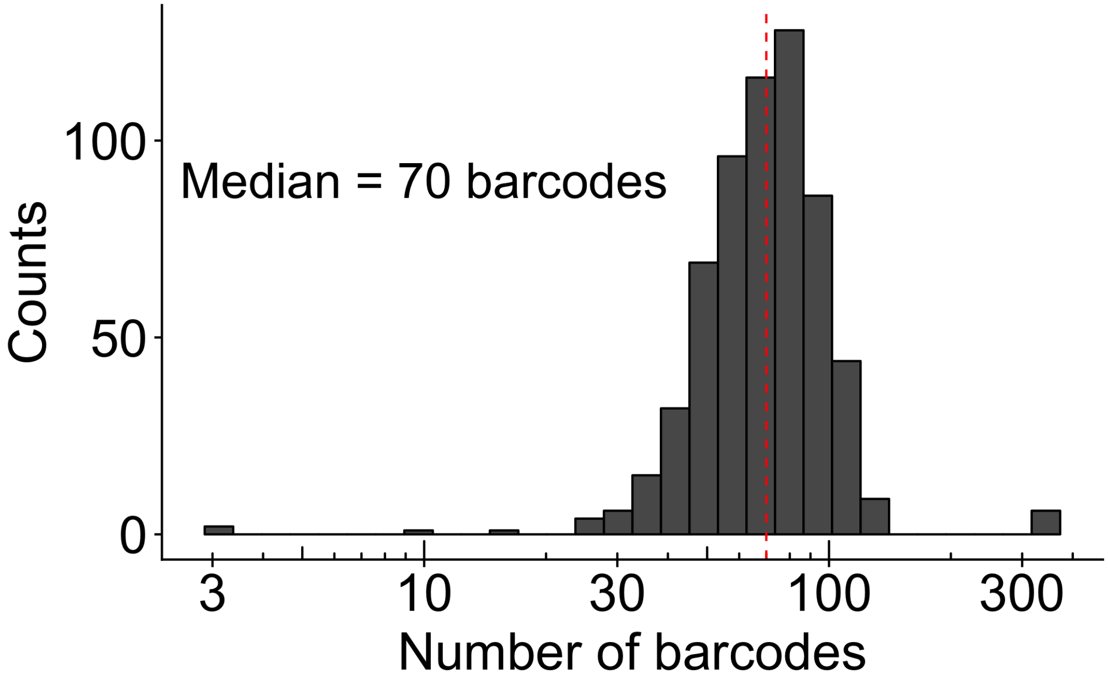

Supplementary Figure 1) Distribution of the number of unique barcodes for operator spacing variants. We recovered promoter activity data for 615 of the 624 (98.6%) variants in the operator spacing library (n = 615, bins = 30). On average, we measured the expression of 70 unique barcodes per variant. Source data are provided as a Source Data file.

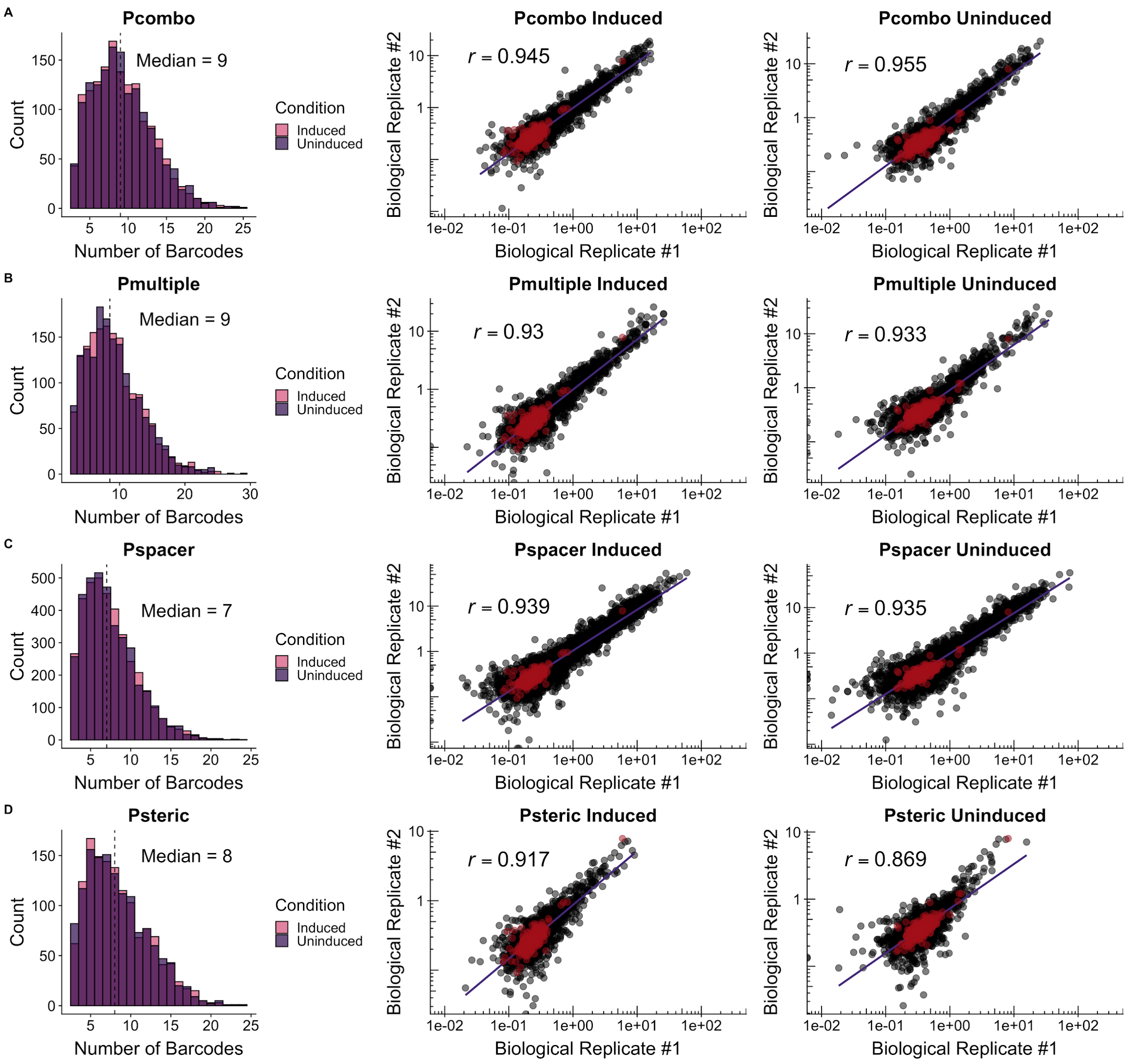

Supplementary Figure 2) Distributions of unique barcode sequences and correlations between biological replicates. Library quality statistics for **a** Pcombo (n = 1493) **b** Pmultiple (n = 1638) **c** Pspacer (n = 3769) **d** Psteric (n = 1369). All variants in each library show strong correlation between biological replicates ($p < 2.2 \times 10^{-16}$, two-sided student's t-test). Red data points indicate the expression of transcriptionally inactive negative controls (described in methods). Source data are provided as a Source Data file.

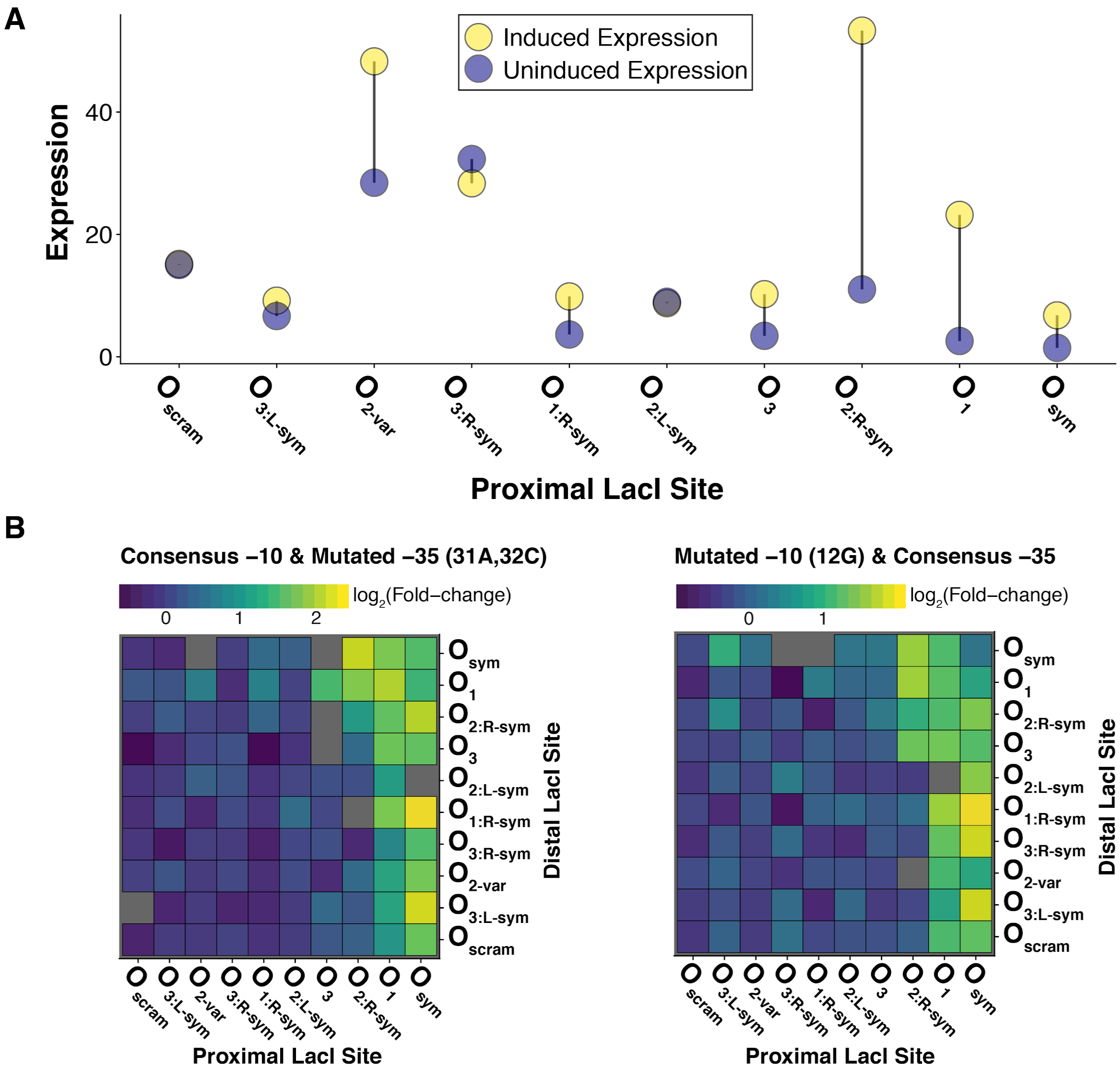

Supplementary Figure 3) Optimal repressor binding sites in the Pcombo library is conditional based on the identity of the core promoter. a Expression ranges of variants containing consensus -10, consensus -35, and distal O_{sym}. Promoters containing O_{sym} in both the *proximal* and *distal* sites have weaker induced expression compared with promoters containing *distal* O_{sym} and a weaker proximal site. **b** Fold-change for Pcombo variants containing one of the consensus -10/-35 elements coupled with a near consensus -10/-35 element. The best operator combination by fold-change differs from that of the consensus core promoter (see Figure 2E) when either the -10 or -35 element is mutated, suggesting an interplay between the repressor sites and core promoter strength. Source data are provided as a Source Data file.

A)

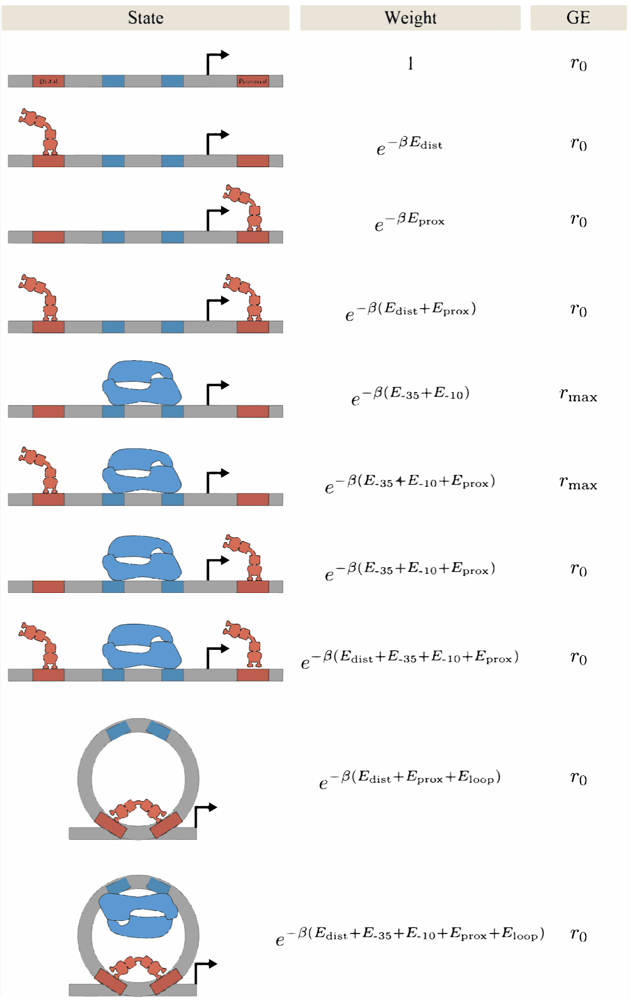

B)

Parameter	Value
r_{min}	0.879
r_{max}	25.14
E_{loop}	−2.49
$p_{act}^{repressor}$	0.0282
$E[lac_{O1}]$	−0.23
$E[lac_{O1}^{rightSym}]$	3.01
$E[lac_{O2}]$	3.91
$E[lac_{O2}^{leftSym}]$	27.88
$E[lac_{O2}^{rightSym}]$	1.58
$E[lac_{O3}]$	2.01
$E[lac_{O3}^{leftSym}]$	3.76
$E[lac_{O3}^{rightSym}]$	25.96
$E[lac_{Oscram}]$	∞
$E[lac_{Osym}]$	−2.09
$E[-10_{cons}]$	0.
$E[-10_{7A}]$	4.
$E[-10_{12A}]$	5.03
$E[-10_{12G}]$	3.77
$E[-35_{cons}]$	−1.96
$E[-35_{30C\_33T}]$	3.58
$E[-35_{31A\_32C}]$	0.66
$E[-35_{33T}]$	1.7

C)

Parameter	Value
r_{min}	0.89
r_{max}	24.09
E_{loop}	−4.66
$p_{act}^{repressor}$	0.001
$E[lac_{O1}]$	−0.23
$E[lac_{O1}^{rightSym}]$	4.68
$E[lac_{O2}]$	5.87
$E[lac_{O2}^{leftSym}]$	∞
$E[lac_{O2}^{rightSym}]$	3.23
$E[lac_{O3}]$	3.65
$E[lac_{O3}^{leftSym}]$	5.61
$E[lac_{O3}^{rightSym}]$	∞
$E[lac_{Oscram}]$	∞
$E[lac_{Osym}]$	−2.21
$E[-10_{cons}]$	0.
$E[-10_{7A}]$	4.1
$E[-10_{12A}]$	5.14
$E[-10_{12G}]$	3.87
$E[-35_{cons}]$	−2.06
$E[-35_{30C\_33T}]$	3.6
$E[-35_{31A\_32C}]$	0.64
$E[-35_{33T}]$	1.7

Supplementary Figure 4) A thermodynamic model for *lacUV5*. a Thermodynamic states of *lacUV5* architecture and their corresponding Boltzmann weights. The probability that the system is in each state is proportional to the relative values of the Boltzmann weights. The system is assumed to elicit a background level of gene expression (GE) given by r_{min} unless RNAP is bound with no repressor bound to the proximal site, in which case a larger level of promoter activity r_{max} is evoked. **b** Best-fit parameter values inferred by fitting this model to the 1,600 promoters with this architecture, simultaneously considering their gene expression with and without 1mM IPTG. **c** Best-fit parameter values inferred when $p_{act}^{repressor}$ at 1 mM IPTG is manually set to its previously reported value 0.001.

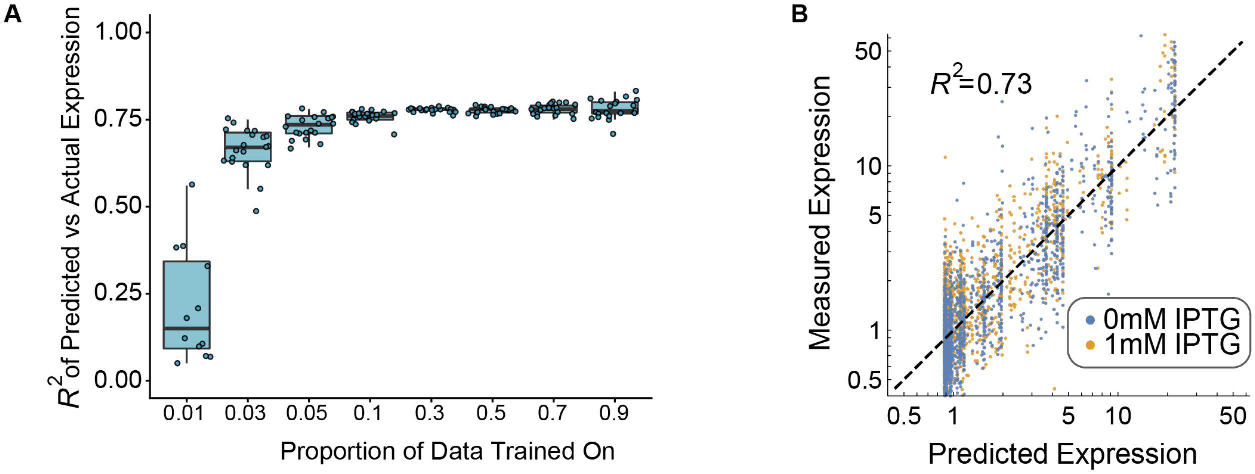

Supplementary Figure 5) Predictive modeling of unseen lacUV5 promoter variants. a Correlation between predicted and actual expression levels when different proportions of data are used as training sets to approximate model parameters. For each proportion, twenty unique, randomized samplings of the Pcombo library were used as training input and the remaining Pcombo promoters were used for prediction. In each boxplot, the lower, middle, and upper hinges correspond to the first quartile, median, and third quartile respectively. Whiskers represent 1.5*IQR from the lower and upper hinges. For proportion = 0.01, eight R^2 values were negative or indeterminate and excluded from the visualization. For all other samples, n = 20. **b** Thermodynamic parameter values fit to the Pcombo library expression (Supplementary Figure 4B) enable moderate ability to predict induced and uninduced expression of Pmultiple variants when used in an adapted thermodynamic model (R^2 = 0.73, $p < 2.2 \times 10^{-16}$, two-sided student's t-test). Source data are provided as a Source Data file.

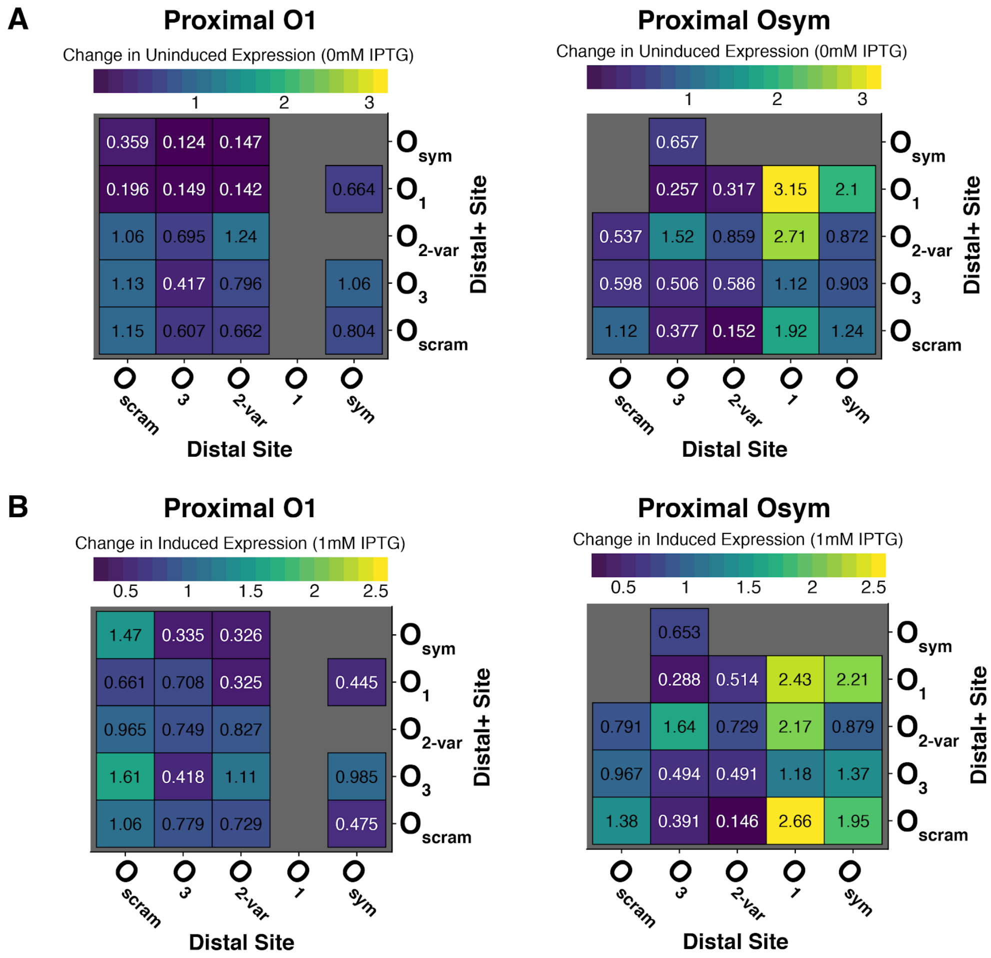

Supplementary Figure 6) Effect of distal+ site on induced and uninduced expression in the Pmultiple library. a Change in uninduced expression of Pmultiple variants with proximal O_1 (left) and O_{sym} (right) relative to their Pcombo counterparts. Distal operator sites are rank-ordered by strength from left to right and Distal+ sites are rank-ordered by strength from bottom to top. **b** Change in induced expression of Pmultiple variants with proximal O_1 (left) and O_{sym} (right) relative to their Pcombo counterparts. Distal operator sites are rank-ordered by strength from left to right and Distal+ sites are rank-ordered by strength from bottom to top. Source data are provided as a Source Data file.

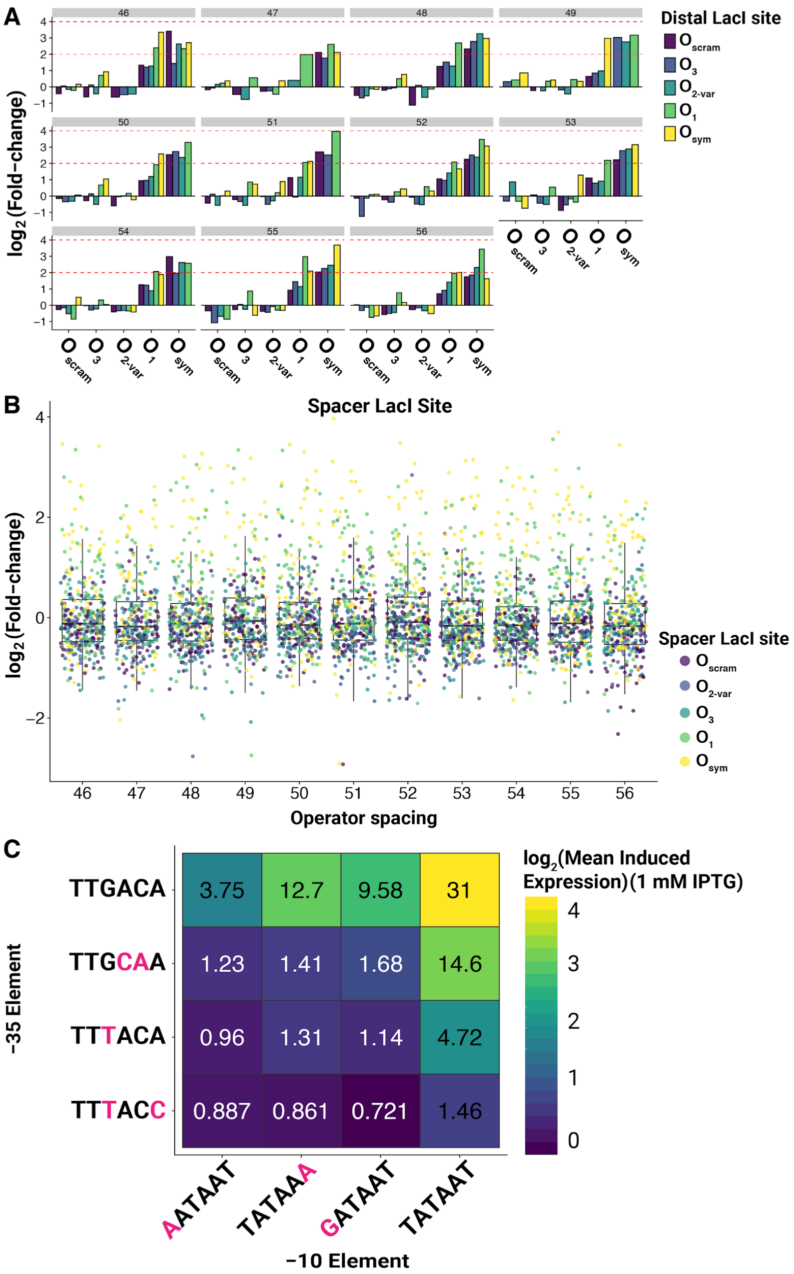

Supplementary Figure 7) Fold-induction and induced expression are modulated by the strengths of repressor sites and the identity of the core promoter in the Pspacer library. a Fold-change of spacer and distal combinations at each operator spacing (ranging from 46 to 56 bp) for Pspacer variants containing the consensus core promoter. **b** Distribution of fold-changes for each distance between operator sites show little effect due to operator distance. Sample sizes for each boxplot are: 46 (n = 347), 47 (n = 296), 48 (n = 341), 49 (n = 317), 50 (n = 351), 51 (n = 337), 52 (n = 364), 53 (n = 358), 54 (n = 368), 55 (n = 342), and 56 (n = 348). In each boxplot, the lower, middle, and upper hinges correspond to the first quartile, median, and third quartile respectively. Whiskers represent 1.5*IQR from the lower and upper hinges. **c** Mean induced expression for each combination of -10 and -35 combinations amongst Pspacer library variants. Source data are provided as a Source Data file.

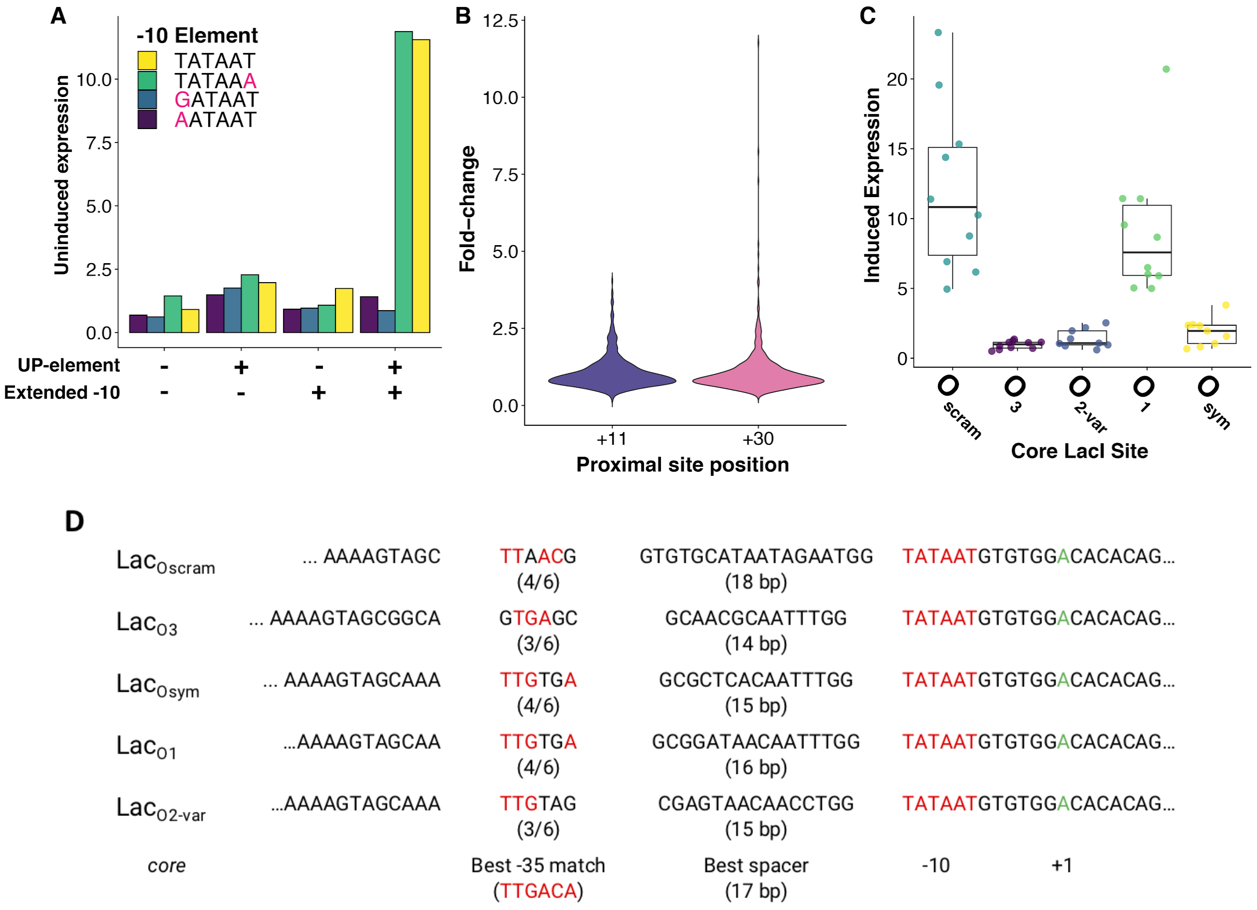

Supplementary Figure 8) Operator site distance and composition influence Psteric architecture viability. a Uninduced expression for promoters containing proximal and core O_{scram} sites, subset by combinations of UP element and extended -10 motifs. **b** Psteric variants with the highest fold-change are observed with a proximal site centered at +30, resulting in a 56 bp spacing between operator sites. Median fold-change between variants with different proximal site positions is similar. **c** Strongly expressed Psteric variants primarily contained core operator sites containing partial matches to the -35 motif, despite not being in the optimal position relative to the -10 motif. Sample sizes for each boxplot are: O_{scram} (n = 10), O3 (n = 9), $O_{2\text{-}var}$ (n = 10), O1 (n = 10), O_{sym} (n = 9). In each boxplot, the lower, middle, and upper hinges correspond to the first quartile, median, and third quartile respectively. Whiskers represent 1.5*IQR from the lower and upper hinges. **d** Potential -35 motif sequence and spacer length for each core operator site. Lac_{Oscram} and Lac_{O1} operator sites contain 4/6 bases matching the -35 motif as well as spacer lengths near the optimal 17 bp. Source data are provided as a Source Data file.

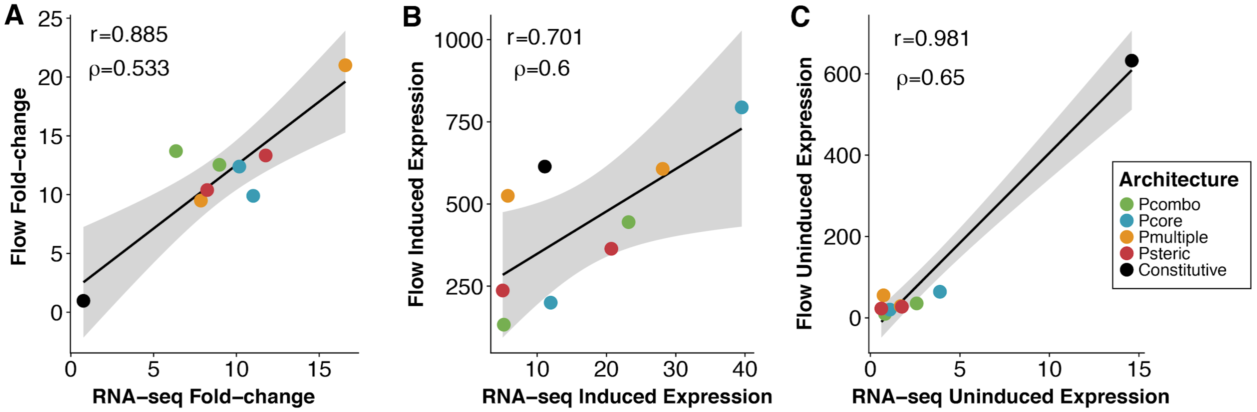

Supplementary Figure 9) Correlations between flow cytometry and RNA-seq. a-c Comparison of RNA-Seq and flow cytometry measurements for promoters individually characterized in **Figure 5.** Strong Pearson correlations are reported between fold-change (r = 0.885, p = 0.001, two-sided student's t-test), induced expression (r = 0.701, p = 0.03, two-sided student's t-test), and uninduced expression (r = 0.981, $p = 3.3 \times 10^{-6}$, two-sided student's t-test) measurements for flow cytometry and RNA-seq. Moderately strong Spearman correlations are also reported between fold-change (ρ = 0.533, p = 0.15, two-sided student's t-test), induced expression (ρ = 0.6, p = 0.10, two-sided student's t-test), and uninduced (ρ = 0.65, p = 0.07, two-sided student's t-test) measurements for flow cytometry and RNA-seq. The shaded region corresponds to 95% confidence interval. Source data are provided as a Source Data file.

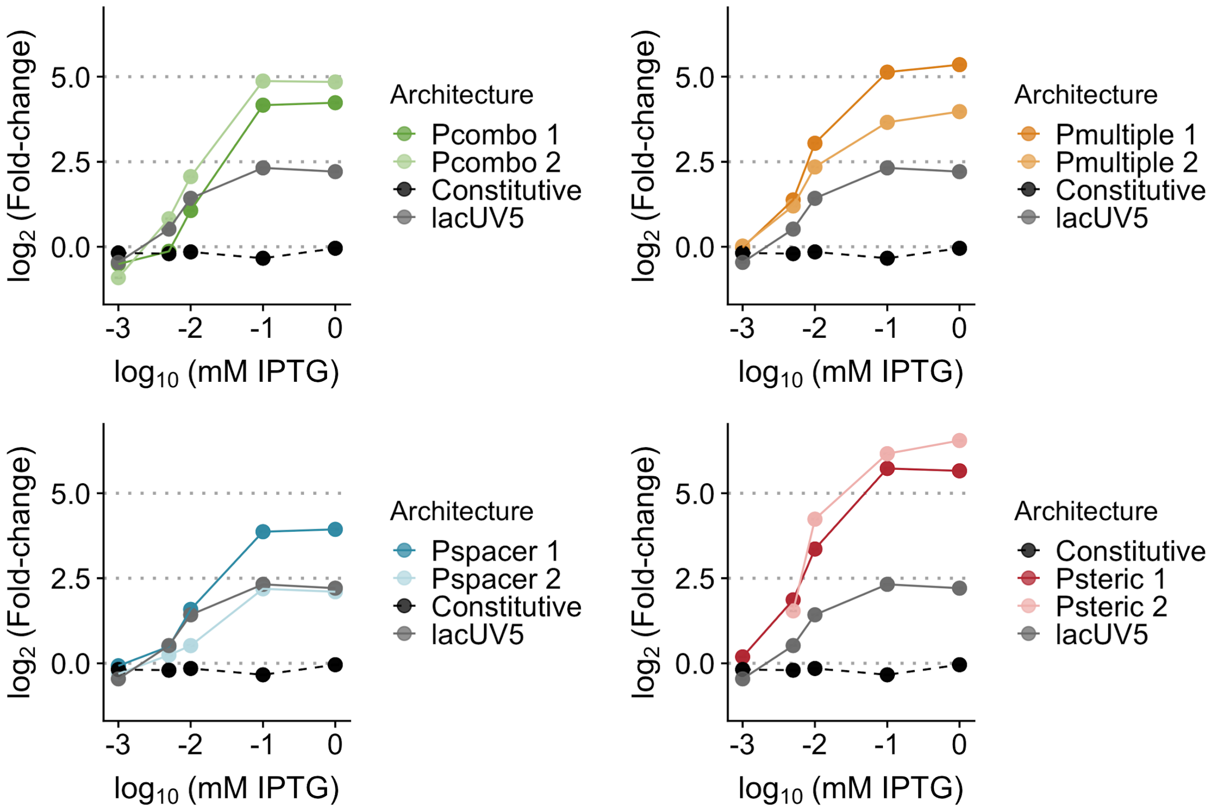

Supplementary Figure 10) Architecture input-output relationships following IPTG induction. Input-output response to IPTG for variants from each architecture compared to *lacUV5* and a constitutively active variant (Constitutive). Data point for 'Psteric 2' at 10^{-3} mM IPTG fell below the limit of detection and was removed from analysis. Source data are provided as a Source Data file.

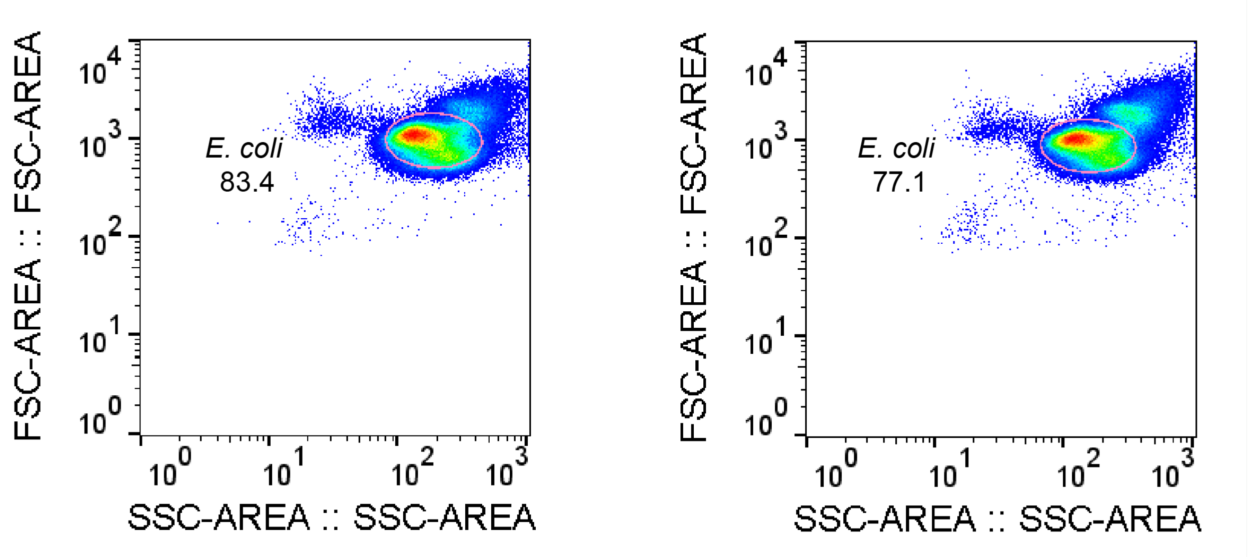

MG1655 Induced
Ungated: 150,000 cells
Gated: 125,145 cells

MG1655 Uninduced
Ungated: 150,000 cells
Gated: 115,640 cells

Supplementary Figure 11) Flow cytometry gating strategy. Gating strategy to analyze *E. coli* cells expressing all promoter variants under induced (left) and uninduced (right) conditions (Figure 5).

TF	*Distal* site (5'>3')	*Proximal* site (5'>3')	*Source Operon*
LacI	G**GGCAGTGAGCGCAACGCAATT**A	G**AATTGTGAGCGGATAACAATT**T	*lacZYA*
GalR	TCTT**GTGTAAACGATTCCAC**TAA	TACCG**GTGGTAGCGGTTACAT**TG	*Lewis et al. 2002*[74]
AraC	GAA**GAAACCAATTGTCCATA**TTG	CCA**TAGCATTTTTATCCAT**AAGA	*araBAD*
PurR	GTTG**AGGAAAACGATTGGCT**GAA	TTT**AAGCAAACGGTGATT**TTGAA	*purA*
GlpR	AAAA**TGTTCAAAATGACGCATG**A	**AAATGGTAAAAAACGAACTT**CAG	*glpTQ*
LldR	**AAGAATTGGCCCTACCAATTCTT**	**CACAATTGGCAGTGCCACTTTTA**	*lldPRD*

Supplementary Table 1) Transcription factor binding sites to test operator spacings. Sequences were acquired from RegulonDB[49] with the exception of the GalR repressor sites which were used in a previous study[74]. Binding sites, represented here in bold font, were appended by the surrounding native sequence context up to 25 bp.

Operator Variant	Sequence(5’>3’)	Inferred Binding energy (K_bT)
O_1	AATTGTGAGCGGATAACAATT	-0.23
$O_{2\text{-var}}$	AAATTGTAGCGAGTAACAACC	3.91
O_3	GGCAGTGAGCGCAACGCAATT	2.01
O_{sym}	AAATTGTGAGCGCTCACAATT	-2.09
O_{scram}	TTAACGGTGTGCATAATAGAA	∞
$O1_{rightsym}$	AATTGTTATCGGATAACAATT	3.01
$O2_{rightsym}$	AAATGTGAGCCGCTCACATTT	1.58
$O2_{leftsym}$	GGTTGTTACTCAGTAACAACC	27.88
$O3_{rightsym}$	AATTGCGTTGGCAACGCAATT	25.96
$O3_{leftsym}$	GGCAGTGAGCGGCTCACTGCC	3.76
O_1-spacer	TTGTGAGCGGATAACAA	---
$O_{2\text{-var}}$-spacer	ATTGTAGCGAGTAACAA	---
O_3-spacer	CAGTGAGCGCAACGCAA	---
O_{sym}-spacer	ATTGTGAGCGCTCACAA	---
O_{scram}-spacer	AACGGTGTGCATAATAG	---

Supplementary Table 2) Operator site sequences for libraries mentioned in Figures 2 - 5. Operator sequences used to assemble libraries with inferred binding energies from the statistical mechanics expression model in Figure 3.

Operator Variant	Sequence (5'>3')	%AT-content
O1-spacer	TTGTGAGCGGATAACAA	58.8%
O2-var-spacer	ATTGTAGCGAGTAACAA	64.7%
O3-spacer	CAGTGAGCGCAACGCAA	58.8%
Osym-spacer	ATTGTGAGCGCTCACAA	52.9%
Oscram-spacer	AACGGTGTGCATAATAG	58.8%
WT lacUV5 spacer	TTTATGCTTCCGGCTCG	47.1%

Supplementary Table 3) Pspacer operator sites and %AT content. LacI operator sites used in the 'Pspacer' library.

-35 element name	Sequence (5'>3')
minus35cons	TTGACA
minus35_31A_32C	TTGCAA
minus35_33T	TTTACA
minus35_30C_33T	TTTACC

Supplementary Table 4) -35 element sequences used in this work. These -35 sequences for RNAP recognition were previously reported and result in a wide range of binding affinities. Non-consensus bases are shown in red.

-10 element name	Sequence (5'>3')
minus10cons	TATAAT
minus10_12G	GATAAT
minus10_12A	AATAAT
minus10_7A	TATAAA

Supplementary Table 5) -10 element sequences used in this work. These -10 sequences for RNAP recognition were previously reported and result in a wide range of binding affinities. Non-consensus bases are shown in red.

UP element name	Sequence (5’>3’)
up_326x	GGAAAATTTTTTTCAAAAGTA
up_136x	GAAAATATATTTTTCAAAAGTA
up_69x	AGAAAATTATTTTAAATTTCCT
no_up	AGCTCATTCATTAGGCACCCCA

Supplementary Table 6) UP element sequences. UP elements used in this work to generate function promoters lacking -35 elements. UP elements are ranked from the top row to bottom by strength.

Primer	Sequence (5’>3’)
GU 59	CATGTTGTCCACTCCAATCGGTGATGGTCCTG
GU 60	GTAATAGCTAAATCCCACCCGATGCCTGCAGG
GU 65	CAAGCAGAAGACGGCATACGAGAT CGAATG CATGTTGTCCACTCCAATCG
GU 66	CAAGCAGAAGACGGCATACGAGAT CTATGC CATGTTGTCCACTCCAATCG
GU 67	CAAGCAGAAGACGGCATACGAGAT GCTAGT CATGTTGTCCACTCCAATCG
GU 68	CAAGCAGAAGACGGCATACGAGAT GTACTG CATGTTGTCCACTCCAATCG
GU 70	AATGATACGGCGACCACCGAGATCTACACGTAATAGCTAAATCCCACCCGATGC
GU 79	CGTGCATAGTGCCATGTTATCCCTGAAGTCGAG
GU 86	CAAGCAGAAGACGGCATACGAGAT GCTAGT CGTGCATAGTGCCATGTTATC
GU 87	CAAGCAGAAGACGGCATACGAGAT GTACTG CGTGCATAGTGCCATGTTATC
GU 89	CATAGCCGAATAGCCTCTCCACC
GU 99	GCGATTGGTCTCACTAGAGCTGTC
GU 100	GGTCAGCCATGGTTATTTGTACAGTTC
GU 102	AATGATACGGCGACCACCGAGATCTACAC
GU 132	TGTCAGGCATATTATCCGCT
GU 133	CGGTTTATGGGTGTTATCGC
GU 134	TCGTATCCCTGCAGGNNNNNNNNNNNNNNNNNNNNGCATGTGAGACCCGGTTTATGGGTGTTATCGC
GU 142	GGTCCAGTGCCATGTTATCCCTGAAGT

Supplementary Table 7) Primers used in the study. See methods for description of primer usage.

Library name	Uninduced expression	Induced expression	Fold-change
Pcombo<br>(N = 1493)	Min: 0.284<br><br>Max: 75.7<br><br>Range: 267x	Min: 0.126<br><br>Max: 57.0<br><br>Range: 453x	Min: 0.222<br><br>Max: 8.97<br><br>Range: 40.4x
Pspacer<br>(N = 3769)	Min: 0.0838<br><br>Max: 193<br><br>Range: 2300x	Min: 0.0567<br><br>Max: 183<br><br>Range: 3230x	Min: 0.132<br><br>Max: 15.6<br><br>Range: 118x
Pmultiple<br>(N = 1638)	Min: 0.0888<br><br>Max: 85.6<br><br>Range: 963x	Min: 0.159<br><br>Max: 74.3<br><br>Range: 467x	Min: 0.174<br><br>Max: 16.6<br><br>Range: 95.2x
Psteric<br>(N = 1369)	Min: 0.164<br><br>Max: 33.3<br><br>Range: 202x	Min: 0.135<br><br>Max: 23.3<br><br>Range: 173x	Min: 0.217<br><br>Max: 11.8<br><br>Range: 54.3x

Supplementary Table 8) Library range statistics. Reported statistics amongst all promoters characterized in each library.

Supplementary Note

We developed a statistical mechanical model of binding that could analyze the thousands of promoter combinations to extrapolate under what conditions optimal fold-change can be achieved. We began by enumerating all possible states available to the *lacUV5* architecture (Supplementary Figure 4A), assigning the relative probability of each state ($e^{-\beta E}$) where E equals the sum of all binding free energies arising in that state due to binding or looping. We assume that all states where RNAP is bound and the proximal LacI site is not bound give rise to gene expression r_{max}, whereas all other states have a small background level of gene expression r_{min}[9,57]. Upon summing the contributions from all states, the steady-state gene expression of a variant is given by:

$$Gene\ expression = \frac{r_{min}(\Phi - e^{-\beta(E_{-35}+E_{-10})}(1+e^{-\beta E_{dist}})) + r_{max}(e^{-\beta(E_{-35}+E_{-10})}(1+e^{-\beta E_{dist}}))}{\Phi} \quad (1)$$

where

$$\Phi = (1 + e^{-\beta(E_{-35}+E_{-10})})(1 + e^{-\beta E_{dist}} + e^{-\beta E_{prox}} + e^{-\beta(E_{prox}+E_{dist})} + e^{-\beta(E_{prox}+E_{dist}+E_{loop})}) \quad (2)$$

represents the partition function (the sum of the Boltzmann weights for all states). This compact form signifies that gene expression only arises when RNAP is bound (and contributes $E_{-35} + E_{-10}$ to the free energy) and the distal LacI site is either unoccupied or occupied (adding free energy 0 or E_{dist}, respectively).

With only slight modification, the above equation for gene expression can also be used to model these same promoters at 1 mM IPTG. In the absence of IPTG, all repressors are in the active state, in which they are capable of binding the promoter[58]. When 1 mM IPTG is added, only a small fraction, $p_{act}^{repressor}$ of these repressors will be active. Hence the Boltzmann weights $e^{-\beta E_{dist}}$ and $e^{-\beta E_{prox}}$ of bound LacI, which are proportional to the number of active repressors, must all be multiplied by $p_{act}^{repressor}$. This can be achieved by modifying:

$$\begin{aligned} E_{dist} &\to E_{dist} - k_B T log(p_{act}^{repressor}) \\ E_{prox} &\to E_{prox} - k_B T log(p_{act}^{repressor}) \\ E_{loop} &\to E_{loop} + k_B\, T\, log(p_{act}^{repressor}) \end{aligned} \quad (3)$$

in the equation for gene expression above, where the last relation for the looping free energy arises because looping corrects for the effective concentration of a singly bound repressor binding with its other dimer. In other words, when the repressor goes from the unbound to the monovalently bound state, and again from the monovalent to bivalently bound state, it gains binding energy from its contact with the DNA. However, the entropic penalty (due to the loss of translational and rotational freedom) of the unbound to monovalently bound transition is far larger than the corresponding loss between the monovalent and bivalent states, and this boost from bivalent binding is accounted for by the free energy of looping. In summary, by introducing the

single additional parameter $p_{act}^{repressor}$, we can extend our characterization of the 1,493 promoters in the absence of IPTG to also include their gene expression at 1 mM IPTG.

Figures 3A,B indicate gene expression aligns with our experimental measurements using the fit value $p_{act}^{repressor}$=0.028. This implies that 28 of every 1000 repressor molecules are in the active state at 1 mM IPTG, or equivalently that each repressor fluctuates sporadically between an active and inactive state but will on average spend 2.8% of the time in the active state. We note that this value for the fraction of active repressors inferred from our data is 28 times larger than a previously imputed value for $p_{act}^{repressor}$=0.001 at 1 mM IPTG[58]. Enforcing this previous value for $p_{act}^{repressor}$ while fitting the model resulted in comparable parameter values (Supplementary Figure 4C) and overall fit (R^2 = 0.79, $p < 2.2 \times 10^{-16}$, two-sided student's t-test).

Extending the model framework for the Pmultiple architecture

To extend our equation for gene expression to predict expression of Pmultiple promoters, we modified our equation for gene expression to consider the additional states that would be possible given the presence of a distal+ LacI site. For simplicity, we assume that LacI cannot be simultaneously bound to both the distal and distal+ site given that both sites are immediately adjacent to one another, and hence relatively few additional states need to be introduced into the model. Below is a comparison of the original and modified equations:

$$Gene\ Expression\ = \frac{r_{min}(Z_{notProx}+Z_{prox}+e^{-\beta(E_{-35}+E_{-10})}Z_{prox})+r_{max}e^{-\beta(E_{-35}+E_{-10})}Z_{notProx}}{(1+e^{-\beta(E_{-35}+E_{-10})})(Z_{Prox}+Z_{notprox})} \quad (4)$$

Where Z_{prox} represents the sum of weights for all states where the proximal site is bound while $Z_{notProx}$ equals the sum of weights for all states where the proximal site is not bound. These states differ between the initial model without a distal+ site and the secondary model which includes a distal+ site. Without a distal+ site, these variables represent:

$$Z_{prox} = e^{-\beta E_{prox}} + e^{-\beta(E_{prox}+E_{dist})} + e^{-\beta(E_{prox}+E_{dist}+E_{loop})} \quad (5)$$
$$Z_{notProx} = 1 + e^{-\beta E_{dist}}$$

With a distal+ site, we must account for the new states possible with this configuration, so these variables represent:

$$Z_{prox} = e^{-\beta E_{prox}} + e^{-\beta(E_{prox}+E_{dist})} + e^{-\beta(E_{prox}+E_{dist+})} + e^{-\beta(E_{prox}+E_{dist}+E_{loop})} + e^{-\beta(E_{prox}+E_{dist+}+E_{loop})} \quad (6)$$
$$Z_{notProx} = 1 + e^{-\beta E_{dist}} + e^{-\beta E_{dist+}}$$

Notably, the equation for gene expression remains unchanged and the only difference is what states are within Z_{prox} (which sums over all states where the promoter is repressed), and $Z_{notProx}$ (where the proximal site is unbound and the promoter will still be active). Specifically, the terms in the modified Z_{prox} represent the states where only the proximal LacI site is bound, the proximal and distal sites are bound, the proximal and distal+ sites are bound, the proximal and distal sites

are bound and loop the DNA, and that the proximal and distal+ sites are bound and loop the DNA. On the other hand, Z_{notProx} has three terms representing that neither the distal+ nor distal sites are bound (1), that only the distal site is bound ($e^{-BE_{dist}}$), and that only the distal+ site is bound ($e^{-BE_{dist+}}$). Relaxing the assumption that LacI could not be bound to both distal and distal+ sites did not affect the resulting R^2.
