# Supplementary material for: Multiplexed characterization of rationally designed promoter architectures deconstructs combinatorial logic for IPTG-inducible systems
Source: Nat Commun. 2021 Jan 12;12:325. doi: 10.1038/s41467-020-20094-3 (PMC7804116; doi:10.1038/s41467-020-20094-3)
Supplement: Supplementary file 2 — Reporting Summary [file 41467_2020_20094_MOESM2_ESM.pdf]

nature research

Corresponding author(s): Guillaume Urtecho (gurtecho@ucla.edu); Sriram Kosuri (sri@ucla.edu);

Last updated by author(s): 10/28/2020

Reporting Summary

Nature Research wishes to improve the reproducibility of the work that we publish. This form provides structure for consistency and transparency in reporting. For further information on Nature Research policies, see Authors & Referees and the Editorial Policy Checklist.

Statistics

For all statistical analyses, confirm that the following items are present in the figure legend, table legend, main text, or Methods section.

n/a	Confirmed	
☐	☒	The exact sample size (n) for each experimental group/condition, given as a discrete number and unit of measurement
☐	☒	A statement on whether measurements were taken from distinct samples or whether the same sample was measured repeatedly
☐	☒	The statistical test(s) used AND whether they are one- or two-sided<br>*Only common tests should be described solely by name; describe more complex techniques in the Methods section.*
☒	☐	A description of all covariates tested
☐	☒	A description of any assumptions or corrections, such as tests of normality and adjustment for multiple comparisons
☐	☒	A full description of the statistical parameters including central tendency (e.g. means) or other basic estimates (e.g. regression coefficient) AND variation (e.g. standard deviation) or associated estimates of uncertainty (e.g. confidence intervals)
☐	☒	For null hypothesis testing, the test statistic (e.g. F, t, r) with confidence intervals, effect sizes, degrees of freedom and P value noted<br>*Give P values as exact values whenever suitable.*
☒	☐	For Bayesian analysis, information on the choice of priors and Markov chain Monte Carlo settings
☒	☐	For hierarchical and complex designs, identification of the appropriate level for tests and full reporting of outcomes
☐	☒	Estimates of effect sizes (e.g. Cohen's d, Pearson's r), indicating how they were calculated

Our web collection on statistics for biologists contains articles on many of the points above.

Software and code

Policy information about availability of computer code

Data collection	Flow cytometry data was gathered using ProSort Version 1.6 (BioRad).
Data analysis	Sequencing data was processed using UNIX and R (Version 3.5.2); Figures were generated in R (Version 3.5.2) and Mathematica (Version 12.0); Custom code used in UNIX, R, and Mathematica for this manuscript are available on Github (https://github.com/timcyu/inducible_architecture); Flow cytometry data was analyzed using FlowJo (Version 10.6.1) and Excel (Version 16.41).

For manuscripts utilizing custom algorithms or software that are central to the research but not yet described in published literature, software must be made available to editors/reviewers. We strongly encourage code deposition in a community repository (e.g. GitHub). See the Nature Research guidelines for submitting code & software for further information.

Data

Policy information about availability of data

All manuscripts must include a data availability statement. This statement should provide the following information, where applicable:

- Accession codes, unique identifiers, or web links for publicly available datasets
- A list of figures that have associated raw data
- A description of any restrictions on data availability

Raw data and promoter expression datasets are available without restrictions through NCBI Gene Expression Omnibus (Accession no. GSE145630).

Field-specific reporting

Please select the one below that is the best fit for your research. If you are not sure, read the appropriate sections before making your selection.

☒ Life sciences ☐ Behavioural & social sciences ☐ Ecological, evolutionary & environmental sciences

For a reference copy of the document with all sections, see nature.com/documents/nr-reporting-summary-flat.pdf

Life sciences study design

All studies must disclose on these points even when the disclosure is negative.

Sample size

To determine the size requirement of the promoter libraries, we computed the number of cells needing to be cloned to achieve ~10x coverage of the library while accounting for errors in synthesized promoter sequences. i.e. for a library of 10,212 variants and expecting 40% error-free oligos, we would attempt to generate (10,212 variants x 10 barcodes per variant / 0.4 rate of error-free oligos) = 255,300 clones minimum to achieve sufficient library coverage. Increasing these number of clones typically improves the robustness of the assay, albeit with diminishing returns. This sample size is sufficient to significantly reduce the variability of median promoter expression measurements between technical replicates.

Data exclusions

No data were excluded from the analysis

Replication

Two biological replicates were performed for all barcode quantification analyses with successful replication. In these cases, separate cultures of the library were grown, RNA/DNA was extracted, and barcodes were prepared for targeted amplicon sequencing. Barcode expression measurements were compared between replicates to ensure robust estimations of promoter activity.

Randomization

This is not relevant to our study. Promoters were measured in a single, pooled group.

Blinding

This is not relevant to our study. Groups were not allocated in this work and conclusions were determined through objective and statistical analyses.

Reporting for specific materials, systems and methods

We require information from authors about some types of materials, experimental systems and methods used in many studies. Here, indicate whether each material, system or method listed is relevant to your study. If you are not sure if a list item applies to your research, read the appropriate section before selecting a response.

Materials & experimental systems

n/a	Involved in the study	
☒	☐	Antibodies
☒	☐	Eukaryotic cell lines
☒	☐	Palaeontology
☒	☐	Animals and other organisms
☒	☐	Human research participants
☒	☐	Clinical data

Methods

n/a	Involved in the study	
☒	☐	ChIP-seq
☐	☒	Flow cytometry
☒	☐	MRI-based neuroimaging

Flow Cytometry

Plots

Confirm that:

☒ The axis labels state the marker and fluorochrome used (e.g. CD4-FITC).

☒ The axis scales are clearly visible. Include numbers along axes only for bottom left plot of group (a 'group' is an analysis of identical markers).

☒ All plots are contour plots with outliers or pseudocolor plots.

☒ A numerical value for number of cells or percentage (with statistics) is provided.

Methodology

Sample preparation

All cells are grown to log phase (OD600 0.3~0.7) and diluted 1:100 in PBS before being passed through a filter cap to remove aggregates or large debris.

Instrument

Biorad S3 Cell Sorter equipped with dual lasers (488 nm & 561 nm)

Software	Flow Cytometry data was analyzed using FlowJo (Version 10.6.1)
Cell population abundance	No cell sorting was performed in this work.
Gating strategy	All populations were individually gated by FSC-Area & SSC-Area to isolate the greatest density of E. coli cells from confounding particles or doublets. Positive cell thresholds were determined by maximizing the density of an ovular boundary manually drawn over a control MG1655 E. coli strain and applying this to all downstream samples.

☒ Tick this box to confirm that a figure exemplifying the gating strategy is provided in the Supplementary Information.
