# Supplementary material for: Multiplexed characterization of rationally designed promoter architectures deconstructs combinatorial logic for IPTG-inducible systems
Source: Nat Commun. 2021 Jan 12;12:325. doi: 10.1038/s41467-020-20094-3 (PMC7804116; doi:10.1038/s41467-020-20094-3)
Supplement: Supplementary file 4 — Source Data [file 41467_2020_20094_MOESM4_ESM.zip › source_data/README.rtf]

Figure1D.txt: Generates Figure 1DFigure1E_top.txt: Generates top panel of Figure 1EFigure1_EFG_S1.txt: Generates bottom panel of Figure 1E, Figures 1F and 1G, and Figure S1Figure2.txt: Generates Figures 2B, 2C, 2D, and 2EFigure3A.csv: Generates Figure 3AFigure3B.csv: Generates Figure 3BFigure3C.csv: Generates Figure 3CFigure3D.txt: Generates Figure 3D (file is called modelExp.txt in script) Figure4A.txt: Generates Figure 4AFigure4B1.txt: Generates left side of Figure 4BFigure4B2.txt: Generates right side of Figure 4BFigure4C.txt: Generates Figure 4CFigure4D.txt: Generates Figure 4DFigure5.xlsx: Generates all of Figure 4FigureS2.txt: Generates all of Figure S2FigureS3.txt: Generates all of Figure S3FigureS4.csv: Generates all of Figure S5 (file is called induce_combo.csv in script)FigureS5A.csv: Generates Figure S5AFigureS5B.csv Generates Figure S5BFigureS6.txt: Generates all of Figure S6FigureS7.txt: Generates all of Figure S7FigureS8_A.txt: Generates Figure S8AFigureS8_BC.txt: Generates Figure S8B and S8CFigureS9.txt: Generates all of Figure S9FigureS10.txt: Generates all of Figure S10
